# Viewpoint-Controllable Telepresence: A Robotic-Arm-Based Mixed-Reality Telecollaboration System

**DOI:** 10.3390/s23084113

**Published:** 2023-04-19

**Authors:** Le Luo, Dongdong Weng, Jie Hao, Ziqi Tu, Haiyan Jiang

**Affiliations:** Beijing Engineering Research Center of Mixed Reality and Advanced Display, Beijing Institute of Technology, Beijing 100081, China; l_luo@bit.edu.cn (L.L.);

**Keywords:** mixed reality, telepresence, human computer interaction, robotic arm

## Abstract

In mixed-reality (MR) telecollaboration, the local environment is remotely presented to a remote user wearing a virtual reality (VR) head-mounted display (HMD) via a video capture device. However, remote users frequently face challenges in naturally and actively manipulating their viewpoints. In this paper, we propose a telepresence system with viewpoint control, which involves a robotic arm equipped with a stereo camera in the local environment. This system enables remote users to actively and flexibly observe the local environment by moving their heads to manipulate the robotic arm. Additionally, to solve the problem of the limited field of view of the stereo camera and limited movement range of the robotic arm, we propose a 3D reconstruction method combined with a stereo video field-of-view enhancement technique to guide remote users to move within the movement range of the robotic arm and provide them with a larger range of local environment perception. Finally, a mixed-reality telecollaboration prototype was built, and two user studies were conducted to evaluate the overall system. User study A evaluated the interaction efficiency, system usability, workload, copresence, and user satisfaction of our system from the remote user’s perspective, and the results showed that our system can effectively improve the interaction efficiency while achieving a better user experience than two traditional view-sharing techniques based on 360 video and based on the local user’s first-person view. User study B evaluated our MR telecollaboration system prototype from both the remote-user side and the local-user side as a whole, providing directions and suggestions for the subsequent design and improvement of our mixed-reality telecollaboration system.

## 1. Introduction

Since the onset of the COVID-19 pandemic, mixed-reality remote collaboration has gained increasing attention. In this type of collaboration, remote users observe the local environment through virtual reality (VR) head-mounted displays (HMDs) and collaborate with local users wearing AR HMDs [1,2,3]. In traditional mixed-reality remote collaboration, the video capture device is typically placed in a fixed position in the local environment [4] or on a local user’s HMD [5], providing a fixed perspective or the local user’s first-person view of the local environment, and remote users cannot actively and freely switch views. In recent research on remote presentations, researchers have mainly deployed video capture devices on wheeled robots [6,7,8] or drones [9,10], allowing remote users to move the viewing perspective by operating these devices. However, these systems have limitations in terms of control freedom and accuracy, which prevent remote users from freely and accurately observing the local environment.

**Figure 1:**
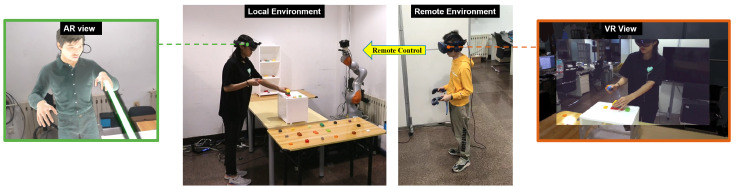
Telecollaboration between a local user wearing an AR HMD and a remote user wearing a VR HMD. A robotic arm with a stereo camera is set up in the local environment. The remote user controls the arm in the local space to change the viewpoint. The local user sees an avatar controlled by the remote user superimposed on the robotic arm.

In this paper, we propose Viewpoint-Controllable Telepresence: a mixed-reality telecollaboration system (Video S1) based on robotic arms that allows remote users to obtain a precisely controllable view in the local space (Figure 1). At the same time, to solve the problem of the limited field of view of the stereo camera and limited movement range of the robotic arm, we combine stereo video with a 3D reconstruction to enhance the field of view of the remote user. When the remote user moves beyond the movement range of the robotic arm, the reconstructed 3D space can continue to provide the remote user with the environmental information of the local space and guide the remote user back to the working range of the robotic arm. Then, we demonstrate a prototype system that incorporates localized agents and nonverbal cues for the remote user on the local-user side, enabling the remote user to better guide the local user. Finally, we conducted two user studies in which remote users guided users in the local space through search and assembly tasks to evaluate our entire mixed-reality telecollaboration system in comparison with two traditional view-sharing schemes: a view-sharing technique based on the local user’s first-person perspective and a view-sharing technique based on panoramic video.

In summary, the main contributions of this paper are as follows:

- We propose a robotic-arm-based controllable telepresence system that allows remote users to control the view of the local space by moving their heads to observe the local environment more actively and naturally.
- We propose a 3D reconstruction method combined with stereoscopic video for a visual field enhancement that guides remote users to move within the range of movement of the robotic arm and provides them with a larger-scale perception of the local environment.
- We demonstrate a prototype of a mixed-reality telecollaboration system and present the results of two user studies comparing a controllable 3D view (robotic-arm-based view-sharing technique) with two traditional view-sharing techniques, and we then evaluate our prototype system.

The remainder of this paper is structured as follows: Section 2 summarizes previous work; Section 3 describes the composition and interactive interface of our system; Section 4 describes user study A for visual sharing technology on the remote-user side; Section 5 describes user study B for evaluating the overall system; Section 6 provides a discussion; and Section 7 concludes the paper and outlines future work.

## 2. Related Work

### 2.1. MR Remote Collaboration System

Applying mixed-reality technologies to remote collaboration can effectively enhance the interaction experience. Wang et al. [11] combined augmented reality (AR) and telepresence technologies to enhance distributed cognition among remote designers. Pejsa et al. [12] reproduced the experience of face-to-face collaboration by using an RGB-D camera to capture local users in 3D and project their life-size virtual copies into a remote space. Gurevich et al. [13] allowed remote users to view information in a physical space through a camera mounted on top of a robotic arm while using a pico-projector to deliver visual communication cues from the remote user to the local user in the form of a projection. In previous studies, remote presentation was performed by projection [12,14] and 3D displays [15] to enhance the observation experience of remote users. Additionally, projectors [16,17] can deliver visual communication cues from remote users to local users. Amores et al. [18] used head-mounted displays and depth cameras to create a system that can immerse the remote user in the first-person view of another user and provide a way for remote experts to provide guidance through 3D gestures and sound. With the development of VR HMD and AR HMD technologies, many mixed-reality telecollaboration efforts began to explore the interaction between remote users using VR and local users using AR. Piumsomboon et al. [19] created a mixed-reality telecollaboration system for remote collaboration between augmented reality (AR) and augmented virtual (AV) users, using the telepresence of the avatar’s head and hands to represent the user. Teo et al. [20,21] proposed an MR telecollaboration system combining 360 and panoramic and 3D reconstructed scenes, conducted a series of user studies, and found that remote users outperformed 3D reconstructions in 360${}^{\circ }$ videos. Piumsomboon et al. [22] proposed a prototype multiscale MR collaboration system that supports two collaboration modes, conventional scale collaboration (CSC) and giant-micro collaboration (GMC), where local users can hold a camera and change the shared view for transmission to remote users. Lee et al. [5] evaluated view-sharing techniques in a one-to-many collaborative MR environment, where a camera is placed on the local user’s AR HMD to capture the workspace for transmission to the remote user, who observes it using a VR HMD. Rhee et al. [4] proposed a remote collaboration platform that transmits a remote VR traveler into a mixed-reality collaborative space (MRC space) to interact with an AR host in another physical space, where a 360${}^{\circ }$ camera in a fixed location in the physical space provides a realistic representation of the environment for the remote user. In these works, the viewpoint of a remote user in the local space is typically either fixed or controlled by local users, making it challenging for remote users to actively and naturally adjust their viewpoints to observe the local space.

Additionally, many studies have used localized agents [23,24] and visual communication cues [25,26] with remote users. Teo et al. [1,2,3] investigated adding light pointing and drawing annotations to gestures as nonverbal communication cues in a real-time 360 panorama-based MR remote collaboration system and found that participating local users were able to perform tasks faster and with fewer errors with the help of visual annotation cues and that having a variety of visual cues significantly improved social presence. Piumsomboon et al. [27] proposed an adaptive avatar with directed gaze and gestures for enhancing remote MR collaboration and evaluated the impact of the avatar in two collaboration scenarios, finding that conveying the necessary nonverbal communication cues improved performance on asymmetric object placement tasks. Kim et al. [28] compared four combinations of nonverbal visual cues (hand only, hand + pointer, hand + sketch, and hand + pointer + sketch) in MR remote collaboration. Jing et al. [29] visualized gaze behavior cues virtually in a 360-degree panoramic mixed-reality telecollaboration system.

### 2.2. Movable Telepresence Robots (MTRs)

Movable telepresence robotics allows a robot to move in a local environment, thus providing a larger local view for remote users. Agarwal et al. [30] used a joystick to operate a telepresence robot to enable remote guidance in an operating room. Nguyen et al. [31] proposed a telepresence wheelchair to transmit panoramic images in real time. Oh et al. [32] proposed a robot-assisted telepresence system with virtual reality (VR) devices in a virtual travel scenario.

The combination of VR technology and telepresence robotics also enables the user to control a robot in an immersive environment [10,33,34,35,36,37]. Peppoloni et al. [38] proposed a robot operating system (ROS) integration framework in which the user controls the motion of a remote robot arm through a wearable device and is provided with 3D visual feedback on the robotic action scene through a Kinect camera and HMD. Martinez-Hernandez et al. [39] proposed a wearable interface consisting of multiple sensory modalities for immersive experiences and telepresence through robots. Our wearable interface consists of visual, haptic, and auditory sensory inputs integrated for interactions with humans and objects in remote environments. Su et al. [40] proposed an integrated mapping scheme for motion and visualization based on a mixed-reality (MR) subspace approach for the intuitive and immersive teleoperation of robotic arm–hand systems. Cambuzat et al. [41] described a platform that enables users to seamlessly monitor the head and eye gaze movements of a robotic platform while maintaining coherence between kinesthetic neural cues and visuomotor feedback. This coherence should facilitate the user’s sense of embodiment and presence in a remote environment.

In immersive virtual reality environments, remote users typically use HMDs to observe the local environment. Kratz et al. [7,42] evaluated the use of a head-mounted device with tracking for remote collaboration in robotic telepresence and concluded that the use of an HMD to control the local view reduced task error rates and improved perceived collaboration success and view quality. Martins et al. [8,43] used a head-mounted display on a control alternative search-and-rescue (SAR) robot, whereby the operator was able to perceive a three-dimensional-corrected image of the robot world transmitted by a pair of stereo cameras on the robot. The head-mounted display was also equipped with an integrated head tracker, which controlled the robot’s motion so that the cameras would follow the operator’s head movements. Illing et al. [6,44] reported a study on the effect of different optoelectronic systems on teleoperation and the use of optional VE scenes and virtual robot models for unmanned ground vehicle (UGV) teleoperation. Aykut et al. [45,46] proposed a 3D 360${}^{\circ }$ telepresence system implemented by a mechanically driven stereo vision system, with a head unit to follow the user’s head movement. Karimi et al. [47,48] designed a telepresence mobile robot platform called MAVI for indoor teleoperation scenarios using a four-wheel drive junction and a stereo vision system, where the user controls the robot in a remote environment, providing a stereo view in all possible directions. Ikei et al. [49] proposed an all-around stereo live camera system in which the orientations of two 360${}^{\circ }$ camera lenses are fixed in world coordinates so that these cameras move with the position of the viewer’s eyes. Chandan et al. [50] developed a mobile telepresence robot (MTR) and equipped it with the ability to analyze 360${}^{\circ }$ scenes; it was capable of directing the user’s attention to the area of interest, allowing the user to efficiently and accurately locate the target object in the remote environment.

Several studies have also explored redirected walking and its thresholds. Piumsomboon et al. [51] proposed a multiscale mixed-reality telecollaborative flight telepresence concept and found that manipulating the interpupillary distance (IPD) significantly affected participants’ size perception. Matsumoto et al. [9] explored vertical gain in reorientation techniques using the virtual reality HMD telepresence of unmanned aerial vehicles (UAVs). However, using drones can result in significant latency and reduced accuracy, as well as concerns related to battery life and safety. Zhang et al. [52,53] proposed a telepresence system based on a 3-degree-of-freedom robot carrying a panoramic camera and using redirected walking (RDW) to enable remote users to freely control the local viewpoint. The virtual translation and rotation thresholds for redirection were also investigated. However, wheeled robots are unable to modify the field of view in the vertical direction, meaning that it is still difficult for a remote user to obtain a natural and accurate viewpoint.

## 3. System Overview

### 3.1. Design

**Figure 2:**
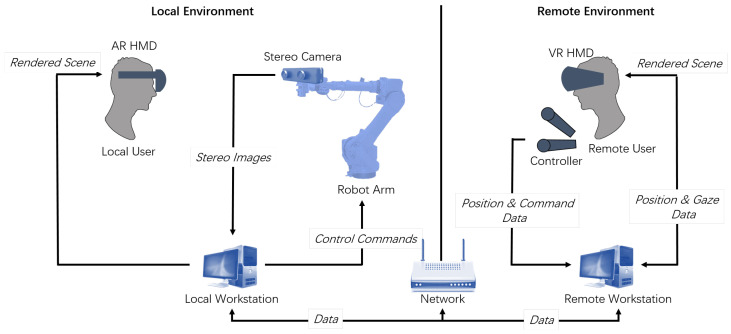
System structure diagram.

We propose a viewpoint-controllable mixed-reality remote interaction system in which remote users can control their viewpoints to naturally observe the local space and collaborate with local users. The local user is able to perceive the remote user through the avatar and receive verbal and nonverbal cues from the remote user. The basic structure is shown in Figure 2.

The remote user wears a VR HMD and observes the local environment through stereoscopic video and 3D models. The robotic arm in the local space is driven by the remote user’s head pose data, and the remote user can also use a pair of controllers to send visual communication cues.

A stereo camera is set on a robotic arm in the local space, and the stereo video is transmitted to the remote-user side in real time over the network. The local user wears an AR HMD to view the real local environment and overlaid augmented information, including the remote user’s avatar and nonverbal cues conveyed by the remote user, which are driven by the remote user’s head, hand, and gaze data.

### 3.2. Field-of-View Enhancement Techniques

The movement space of the robotic arm is limited, and when the remote user moves beyond the working range of the robotic arm, the stereo camera alone cannot provide the local environment perception at the corresponding viewpoint; when the user’s movement and vision do not match, it is easy to produce simulator sickness [54]. At the same time, the field of view of the stereo camera is limited, and the user cannot obtain a larger field of view when wearing the HMD for viewing. To solve these problems, we propose a field-of-view enhancement technique that combines stereoscopic video and 3D scenes. With reference to studies on the human visual system and peripheral vision [51], the center of the visual field below 2${}^{\circ }$ eccentricity is called the foveal visual field, which has the highest visual acuity, and the visual field with approximately 8${}^{\circ }$ eccentricity around the central concave field is called the central visual field. The area that deviates from the central visual field (2${}^{\circ }$ eccentricity) is called the peripheral visual field.

**Figure 3:**
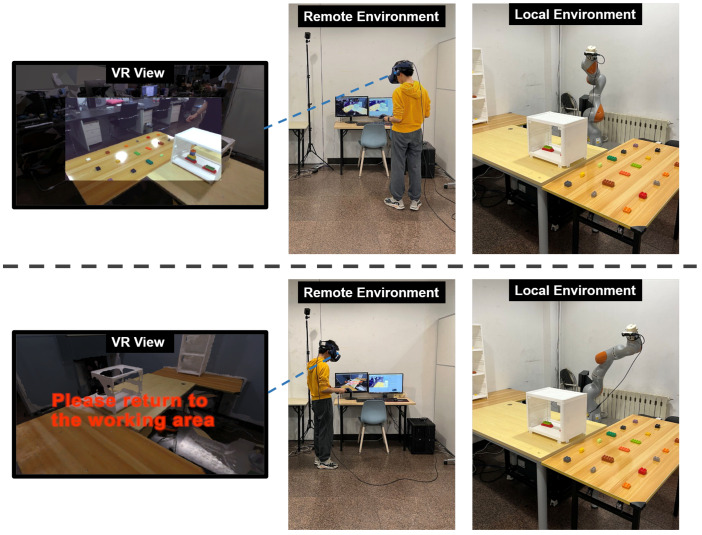
Remote user controlling the robotic arm to observe the local space, where the views from left to right represent the remote user’s perspective, the user position, and the robotic arm position. The top images illustrate the robotic arm operating within its working range, while the bottom images show the robotic arm exceeding its working area.

When the remote user’s viewpoint is within the working range of the robotic arm, the perception of the local environment for the remote user is composed of real-time stereo video captured by the stereo camera and the local 3D scene, with the stereo video displayed in the user’s central field of view and the local 3D scene presented in the peripheral visual area. However, when the user moves beyond the working range of the robotic arm, the stereo video will be hidden, and the user will only be able to see the local 3D scene, as shown in Figure 3. During this time, the robotic arm will still follow the user’s movement within its maximum working range, and the stereo camera will continue to operate, but the stereo video display on the remote user’s end will be hidden to avoid the issue of visual and perceptual mismatch. The stereo video will reappear only once the user has returned to the working area and the robotic arm has synchronized with the user again.

The local 3D scene can be either a pregenerated static model or a real-time reconstruction generated from the stereo images and depth data captured by the local stereo camera, depending on the requirements of different interaction scenarios [55,56]. The reconstructed scene can be updated at a lower real-time rate, which reduces the demands on the processor and data bandwidth.

### 3.3. Interaction Interface for Remote Users

The remote user wears a VR HMD and is placed in a virtual space. The stereoscopic video is displayed in the user’s central field of view through a plane that is fixed in front of the user, while the edge field of view is filled by a 3D reconstruction of the local space (Figure 3). In addition, the remote user uses a pair of handheld controllers, which are displayed synchronously in the HMD, and the remote user can use the handheld controllers to create a virtual ray that conveys pointer information to the local user.

Remote users can control the viewpoint by moving their heads to observe the local space. When the remote user moves beyond the working range of the local robotic arm, the real-time updated stereo video is hidden; the user can only observe the local environment through the reconstructed 3D space but can continue to roam in it. At the same time, a prompt message will appear in the remote user’s field of view, indicating that the remote user has moved beyond the working range of the robotic arm and guiding the user to return to the working area of the robotic arm. When the remote user returns to the working range of the robotic arm, the display of the stereo video turns on and continues to provide the remote user with a real-time stereo view of the local space.

### 3.4. Avatar and Visual Communication Cues

**Figure 4:**
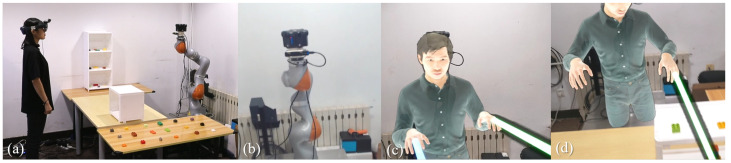
(**a**) The local user and the robotic arm and camera installed in the local environment. (**b**) The real environment in the local user’s view. (**c**,**d**) The avatar and rays superimposed on the robotic arm in the local user’s view through AR.

Referring to a study by Pakanen et al. [23], we selected a high-realism full-body avatar as the remote user’s avatar to be rendered in the local user’s AR HMD (Figure 4b). We calibrated the coordinate system of the remote user space with the local space to display the remote user’s avatar superimposed on the robotic arm position and follow the remote user’s motion. We captured the positional information of the remote user’s head and hand nodes through the VR HMD and a pair of handheld controllers and drove the avatar’s motion through inverse kinematics (IK).

In addition to the remote user’s avatar, we provided visual communication cues for the local user so that the remote user could help the local user perform the task more efficiently and accurately (Figure 4c). We created a vector-direction projected ray from the location of the controller held by the remote user in the local space, determined the direction of the ray projection based on the controller orientation, and then rendered it in the local augmented reality (AR) HMD, where the virtual ray emanated from the hand of the remote user’s avatar in the local user’s field of view. The hiding and display of the ray were controlled by the remote user.

## 4. User Study A: Comparison of Three View-Sharing Technologies

In user study A, we compared our system with the view-sharing techniques of traditional mixed-reality remote systems. We mainly compared two view-sharing schemes, a first-person-perspective view-sharing scheme with a camera above the local user’s head and a view-sharing scheme with a panorama at a fixed location in the local space, similar to the remote collaboration platform proposed by Rhee et al. [4]. Based on our main research questions and set conditions, we propose the following hypotheses:

**H1.**  *The controllable 3D view yields the highest efficiency of task execution.*

**H2.**  *The controllable 3D view yields the highest level of coexistence.*

**H3.**  *The controllable 3D view yields the highest system usability score.*

**H4.**  *The controllable 3D view yields the lowest workload.*

**H5.**  *In terms of simulator sickness performance, the first-person view produces the most sickness, and the controllable 3D view and the panoramic system produce comparable levels.*

### 4.1. Materials

#### 4.1.1. Setup

**Figure 5:**
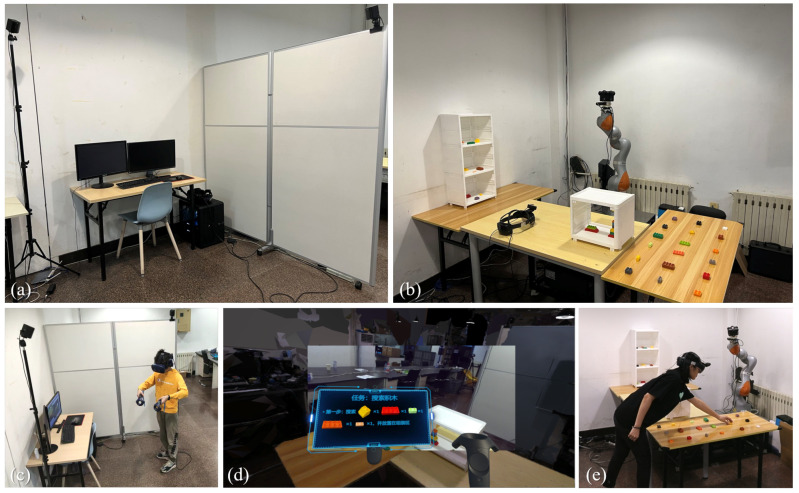
(**a**) The remote space. (**b**) The local space. (**c**) The remote user performing a task. (**d**) The perspective of the remote user, who is viewing the task (The Chinese text in the figure indicates: the current task is to search for the specified blocks and place them in the designated area). (**e**) The local user picking up building blocks.

Our experiments took place in two separate rooms: a remote space with dimensions 2.5 m × 2.5 m and a local space with dimensions 3 m × 2.5 m. The remote space was mostly empty, and the local space contained basic office furniture and objects for which the user needed to search (puzzles, blocks, etc.). The two rooms were separated by a wall, and the user in the remote space could not see the environment in the local space (Figure 5a,b).

**Figure 6:**
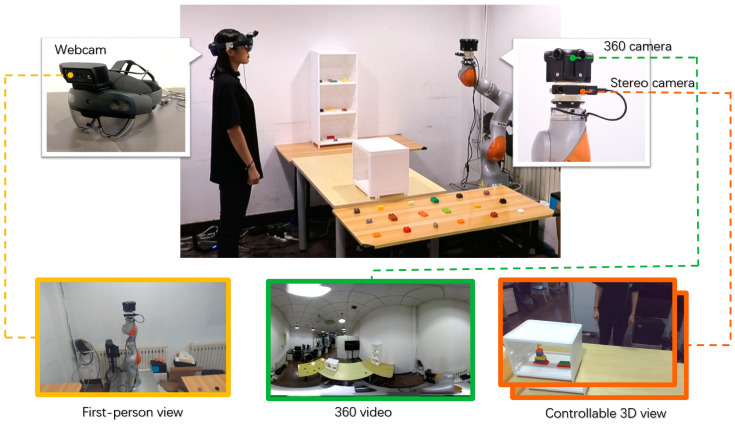
The 3 view-sharing approaches and their implementation in the local space.

In the local space, a Kuka LBR iiwa 14 R820 robotic arm was placed in the scene. The robotic arm was equipped with a ZED mini stereo camera. The local user wore a HoloLens2, and the remote user’s avatar was rendered using Unreal Engine 4.26. In addition, in the first-person view-sharing scenario, we added a C920 webcam to the local user’s HoloLens2. In the panoramic view-sharing scheme, we set up a Kandao Obsidian R panoramic camera (3840 × 2160, 30 FPS) in the local space, and to keep the view as similar to that of the robotic arm as possible, we mounted the panoramic camera directly onto the robotic arm; in this mode, the robotic arm carries the panoramic camera to stay under a height of approximately 1.6 m and stops moving to simulate the fixed view of the local space. All devices in the local space were connected to one device (Intel Core i9-7900X, NVIDIA GeForce RTX3080, 32 GB RAM and 1 TB SSD) (Figure 6).

In the remote space, the user wore an HTC Vive Pro Eye to observe images of the local environment and held a pair of matching controllers, with the user’s head and controller positions tracked in a pair of universal world coordinate systems established by a Lighthouse transmitter. We used the camera to take pictures of various angles of the local space in advance and used Reality Capture to generate 3D models that were rendered on the remote-user side. We used Unity3D 2020f3 to develop our system, with the entire remote side running on a PC (Intel Core i7-6850K, NVIDIA GeForce GTX1080Ti, 32 GB RAM, and 1 TB SSD).

#### 4.1.2. Stimuli

We designed three tasks for the user study in which remote users were required to collaborate with local users to complete a series of tasks, including search, disassembly, and assembly. Since we focus on the comparison of view-sharing techniques for remote users, we let participants play the role of remote users only, while the local user was played by the experimenter. The local user did not initiate any action unless instructed to do so by the remote user. The instructions were mainly given verbally.

The first task was to search the local space for specified LEGO blocks and place them in a specified area, for example, to search for red cubic blocks and place them in area A.

The second task was to search for specified LEGO blocks in the local space and assemble them in the assembly area as required, for example, to search for blue rectangular blocks and red cubic blocks, bring the blocks to the assembly area, and then install the red cubic blocks on the blue rectangular blocks.

The third task was to disassemble a block from a set of assembled LEGO block towers and search the local space for a specified LEGO block to replace it, for example, to disassemble the blue block from the third level of a block tower and the green block from the fifth level and search for two red cubic blocks to replace them.

The task description, which included text and images, was only told to the remote user at the beginning of the experiment and could be viewed by the remote user at any time in VR by using the controller to activate the task description UI (Figure 5d). The local user (experimenter) needed to wait for instructions from the remote user and did not actively behave according to the local task description.

The experiment adopted an in-group design. Each participant experienced all 3 view-sharing techniques and 3 tasks, and participants experienced each view-sharing technique and each task once. We used a 3×3 Latin square to balance the distribution between each group of view-sharing techniques and tasks, and the order of the view-sharing techniques and tasks was randomized relative to the participants.

### 4.2. Experimental Design

#### 4.2.1. Participants

We recruited 24 participants (12 M, 12 F), aged 21–32 years (M = 25.25, SD = 3.08), recruited through the internet, and all of them had normal vision. We asked the participants about their previous VR experience. Three participants had no experience with VR at all, twelve participants had experience with VR but fewer than 10 times, and nine participants had more than 10 experiences with VR. We gave each participant 80 RMB as an experimental reward.

#### 4.2.2. Experimental Process

The experiment began with participants filling out a demographic questionnaire, and then the experimenter explained the purpose of the experiment and the task to the participants, after which the participants entered the remote space of the remote user and began the first round of the experiment.

At the beginning of each round, participants completed the Simulator Sickness Questionnaire (SSQ) pre-experiment scale and then entered the VR space wearing a VR HMD and holding a controller. Participants spent an average of 5 min familiarizing themselves with the operation and interface. When the participants were ready, we set their tasks; the timing started, and the participants began to guide the local users to complete the tasks. When the task goal was achieved, the timer stopped. The participants removed their helmets and completed the questionnaire for that round of the experiment. Once the questionnaire was completed, the participants took a 10 min break while the room and hardware were reset and prepared to start the next round of the experiment.

When all three rounds of the experiment were completed, we administered an exit survey to participants to collect their preferences for the three view-sharing technologies and interviewed them about their comments on the system features and tasks. Overall, the entire experiment lasted an average of one hour.

#### 4.2.3. Measurements

To evaluate the performance of the view-sharing techniques, we used the following metrics.

Task Completion Time: We collected the completion time of each task, which started when the user received the task description and ended when the task was completed.

Number of Instructions: We recorded the number of valid instructions issued by the remote users (some repetitive conversations and repeated confirmations due to everyday language were not recorded), and this metric was used to measure the efficiency of communication.

Social Presence: We used the Networked Minds Social Presence Inventory [57] scale to measure social presence in each condition; the scale consists of six subscales: copresence, attentional allocation, perceived message understanding, perceived affective understanding, perceived affective interdependence, and perceived behavioral interdependence. All rated items were answered on a 7-point Likert scale. We also used the mean of all the subscale scores as the aggregated social presence score (aggHSP) [24].

System Usability: We used the System Usability Scale (SUS) [58] to evaluate the usability of the system for each condition; this metric has a 5-point scale, where a score of 1 means “completely disagree” and a score of 5 means “completely agree”. Users select the appropriate score level for each question based on their subjective perceptions. The odd-numbered questions on the scale are scored as the original score minus 1, and the even-numbered questions are scored as 5 minus the original score, with the final total of the scale being the sum of all question scores multiplied by 2.5.

Workload: We used the NASA-TLX [59] to evaluate the workload of users in each condition; the NASA-TLX scale uses a 7-point Likert scale, where a score of 1 means “totally disagree” and a score of 7 means “totally agree”. The final results are divided into 21 ratings.

Simulator Sickness: We used the SSQ scale [60] to measure user motion sickness in each condition.

Preference: In the exit survey, we asked users to rank each view-sharing technology in order of preference.

### 4.3. Results

We used the IBM SPSS tool to analyze our results, and all pairwise comparisons of the results were subjected to Bonferroni correction.

#### 4.3.1. Task Performance

**Figure 7:**
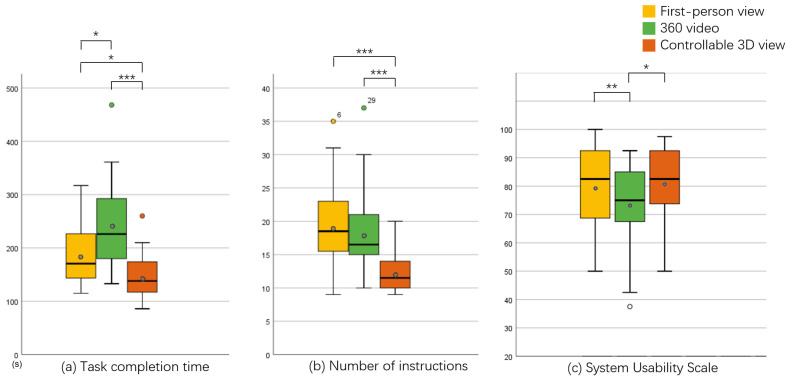
(**a**,**b**) Results of task performance. (**c**) Results of the system usability scale (*: *p* < 0.05, **: *p* < 0.01, ***: *p* < 0.001).

We used two metrics to measure task performance under different conditions, namely, task completion time and the number of instructions issued by the remote users, and the results are shown in Figure 7a,b.

For the task completion time, we used a one-way repeated-measure ANOVA, which showed that the three conditions of first-person view, 360 video, and controllable 3D view differed significantly in task completion time (F(2,46)=16.203, p<0.001). Analyses with pairwise comparisons showed that significant differences existed between the conditions of first-person view and 360 video (p=0.036), first-person view and controllable 3D view (p=0.023), and 360 video and controllable 3D view (p<0.001).

Regarding the number of instructions, we used a one-way repeated-measure ANOVA, which showed that the three conditions of first-person view, 360 video, and controllable 3D view differed significantly in task completion time (F(2,46)=16.799, p<0.001). Analyses with pairwise comparisons showed that no significant differences existed between the first-person view and 360 video conditions, while significant differences existed between the remaining pairs of conditions, namely, first-person view and controllable 3D view (p<0.001) and 360 video and controllable 3D view (p<0.001).

On average, participants took less time to complete tasks in the controllable 3D view and issued fewer instructions than in the first-person view and 360 video.

#### 4.3.2. Social Presence

**Figure 8:**
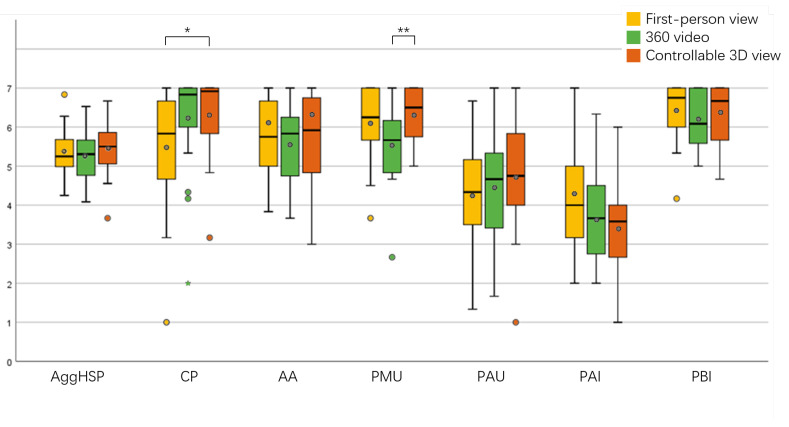
Results for the aggregated social presence and social presence subscales (*: *p* < 0.05, **: *p* < 0.01).

The results for the sense of social presence are shown in Figure 8. Based on our results, there were no significant differences in aggregated social presence scores across the conditions, but there were significant differences in copresence (F(1.516,34.86)=4.753, p=0.023) and perceived message understanding (F(2,46)=7.453, p=0.002) between conditions. Pairwise comparison analysis revealed significant differences in copresence (p=0.025) between the first-person view and controllable 3D view and in perceived message understanding (p=0.001) between the 360 video and controllable 3D view, and no significant differences existed between the remaining conditions.

#### 4.3.3. System Usability

The system usability results are shown in Figure 7c. The Shapiro–Wilk test for these data showed significant deviations from normality, so we performed a Friedman nonparametric test, which showed significant differences in the total system usability scores for the three conditions ($\chi^{2}\left(2\right)=10.14,\;p=0.006$). Paired comparison analysis showed significant differences between the first-person view and the 360 video (p=0.006) and between the 360 video and controllable 3D view (p=0.014). The system usability of 360 video was significantly lower than that of the other two conditions.

#### 4.3.4. Workload

**Figure 9:**
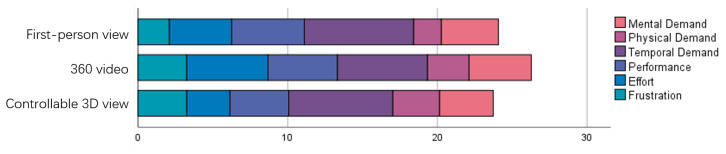
Results for the NASA-TLX scale.

The workload results are shown in Figure 9, where participants had the lowest workload for completing the task in the controllable 3D view and the highest workload for completing the task in the 360 video condition. No significant differences existed between conditions.

#### 4.3.5. Simulator Sickness

We evaluated the SSQ pretest and posttest scores for each view-sharing technique. The statistical results are shown in Table 1. The Shapiro–Wilk test for these data showed significant deviations from normality, so we performed a Wilcoxon signed-rank test, which showed significant differences between the pretests and posttests for all three techniques.

Then, we analyzed the indicators of motion sickness for each condition, including nausea (N), oculomotor (O), disorientation (D), and total score (T). The results are shown in Table 2. The Shapiro–Wilk test for these data showed significant deviations from normality, so we performed a Freedman nonparametric test, which showed that there were no significant differences between the conditions.

#### 4.3.6. Preferences

**Figure 10:**
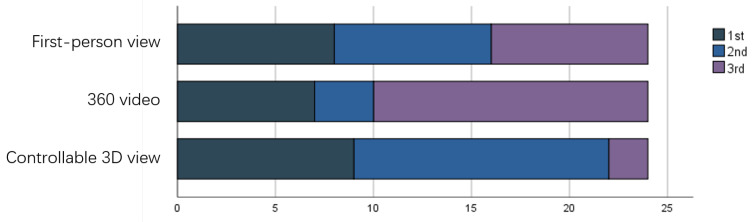
Results for the preference rankings.

The results of the preferences are shown in Figure 10, with an average ranking of 2 for the first-person view, 2.29 for the 360 video, and 1.71 for the controllable 3D view. The controllable 3D view was preferred by more participants.

## 5. User Study B: Evaluating the Proposed MR Telecollaboration System

In user study B, we evaluated the prototype of our mixed-reality telecollaboration system. We gave users a complete experience of the system, including the remote-user side and the local-user side. Then, we collected feedback from the users.

### 5.1. Materials

#### 5.1.1. Setup

The set up was the same as in user study A.

#### 5.1.2. Stimuli

In user study B, we aimed to have participants experience the local-user side and remote-user side of the prototype separately and then collected their evaluations and invited them to suggest improvements. The contents of the experiences were as follows.

Local user perspective:

1. Hiding/showing the local avatar of the remote user.
2. Hiding/showing the indicator ray controlled by the remote user.
3. Observing the performance of the local user’s avatar and the indicator ray under the three visual sharing conditions of Experiment A (in the first-person view, the local user cannot see the participant, and the ray is emitted from its own angle; in the 360 video, the remote user’s avatar cannot move and can only rotate in place; and in the controllable 3D view, the avatar follows the robot arm to move and rotate).

Remote user perspectives:

1. The three viewpoints in user study A.
2. The preestablished model of the local space is either displayed in the controllable 3D view or not.
3. The user interface is displayed when moving out of the working range of the robotic arm.

### 5.2. Experimental Design

#### 5.2.1. Participants

We invited the users in user study A with more than 10 VR experiences to participate in user study B. Five participants (3 M, 2 F) aged 21–32 years (M = 28.4, SD = 4.83) participated in user study B. Two of them had been involved in VR or AR application design or development work.

#### 5.2.2. Experimental Process

First, the participants were guided by the experimenter to experience the remote user perspective and the local user perspective, and the participants experienced the items listed in Section 5.2 in turn. While the participants experienced one of the perspectives, an experimenter would play the role of the user on the other side.

The participants then had the freedom to experience the various features of the experimental system and to try to collaborate with the experimenter in the other role to complete the simple tasks of searching for, assembling, and disassembling blocks. These tasks were not explicitly targeted and were not timed but were designed to allow participants to fully experience the entire telecollaboration system.

After the participants fully experienced both roles, we conducted a semistructured interview with the participants, asking them about their evaluation of the overall system functionality and setting up some hypothetical scenarios and asking them to give their opinions.

## 6. Discussion

### 6.1. User Study A

Overall, our experimental results support H1 well, with the controllable 3D view performing better in the task; the task completion time was significantly shorter, and the remote user issued fewer instructions. This is because, in the first-person view, the remote user needs to issue more movement instructions to the local user, such as “move to the right-hand side” and “look up at the second level of the shelf”, while in the 360 video view, it is more difficult for the remote user to see the shapes of the blocks, and it usually takes longer for the remote user to confirm that the blocks match the task description or even for the local user to bring the blocks closer to the camera so that the remote user can look closely.

In terms of social presence, we can partially accept H2. The aggHSP for the controllable 3D view was higher than that for the other two conditions, but the difference was not significant. In terms of copresence (CP), the controllable 3D view scored significantly higher than the first-person view, with participants citing the “inability to see the partner” in the first-person view as the main reason for this score. In terms of perceived message understanding (PMU), the controllable 3D view scored significantly higher than the 360 video, with seven participants explicitly stating that the greater latency of the 360 video made it appear that “the partner was slower to understand the instructions”.

In terms of system usability, the 360 video scored significantly lower than the controllable 3D view, with seven participants citing “low pixel count and high chromatic aberration” and “high latency” and four participants citing “viewpoint cannot be moved” as influences on the system usability score. There was no significant difference between the first-person view and the controllable 3D view; therefore, we can partially accept hypothesis H3.

In terms of workload, we had to reject H4; although the average workload of the controllable 3D view was lower, it was not significantly different from the other two, and participants generally felt that the workload was mainly related to the specific task settings used and the form of interaction, which did not cause much difference between the views.

In terms of simulator sickness, we had to reject H5; participants experienced mild sickness in all three conditions, but there were no significant differences between the conditions. Participants in the first-person view did not exhibit higher sickness symptoms as we had assumed, probably because our experiment took place in a smaller space where local users did not move particularly fast and because the experiment was not long enough. Users can tolerate a milder mismatch between visual and physical motion.

In the exit survey, we asked users to rank their preferences for the three visual sharing technologies and asked them why. Eight users preferred the first-person view because they thought it was “more immersive and intuitive to give instructions” and “the perspective is the same as the partner’s, so it is easier to communicate without having to change orientation”. Seven users liked the 360 video the most because “the field of view is wider”, “you can determine the approximate location of the blocks faster and quickly direct your partner to get the blocks”, and “the sense of vertigo is minimal”. Nine users liked the controllable 3D view the most because “you can actively control your own viewpoint and observe the whole space more freely” and “you can observe the blocks that are blocked more clearly, and the blocks that you can’t see can be moved closer to observe them by themselves”.

### 6.2. User Study B

Four of the five participants thought that adding an avatar for the local user was a great design, and only one participant thought that the avatar was optional: “I don’t think the avatar will affect the overall progress of the experiment because it doesn’t replace the local user for physical actions, and the instructions given by voice and ray are sufficient”. Two users suggested improvements to the avatar, reporting that “the avatar needs to be more natural when moving” and “I would like to add more expressions and movements, as the current expressions and movements look stiff”.

All five participants agreed that adding the ray improves the efficiency of the interaction and enhances the interaction experience. Two participants reported the following: “In the previous experiment (user study A), I had to think about my orientation in relation to my partner, but it would have been easier to have the ray so that I could have told my partner to go get the block indicated by the ray”; “Using the ray would have made it clearer to show my partner the area I wanted him to focus on. It is convenient to use the ray to show my partner the area I want him to focus on”. One participant suggested that “if the end of the ray was on the object, it would be more accurate in conveying information”.

Regarding the use of the static model as a visual field enhancement, two participants found it useful. The participants reported that “the 3D model allows me to get a larger field of view and to easily return to the work area after losing the live view beyond the work area”. Two participants felt that improvements were needed and reported that “the reconstructed model is relatively rough, which ruins my immersion” and “the edges of the model and the video screen sometimes don’t match properly and look a little strange”. One participant suggested the possibility of using the 360 video as a field-of-view enhancement solution but reconsidered when we showed her the mismatch between the movement and field of view due to the delay of the 360 video.

In terms of the three view-sharing techniques, the reports of the five participants were generally consistent with those in Experiment A. In particular, one participant preferred the first-person view in user study A, but after experiencing the local-user side, she thought that, in the first-person view, “the local user could not see the remote user’s avatar and could not perceive the partner’s behavior and state, so it was better to have interaction with the avatar”.

### 6.3. Implementation Guidelines

We summarize the advantages and disadvantages of the three perspective-sharing techniques in the use of this system based on user feedback in user studies A and B, as shown in Table 3.

In scenarios that require the remote user to move the viewpoint (e.g., finding a book on a multilevel bookshelf) or in which there is an obscuring relationship between items (e.g., finding a specific spice in a kitchen spice rack), the controllable 3D view is clearly more suitable. Even in tasks that can be accomplished in a first-person or panoramic view, the ability of the remote user to actively control the change in view by moving his or her head can improve the user interaction experience and give the user a greater sense of immersion.

In our system prototype, we have actually combined a first-person view, a 360 video, and a controllable 3D view. We think it is possible to use 360 video as a field-of-view enhancement solution if a lower-latency 360 video solution is available. However, the 3D reconstructed local environment model still has its advantages. When the remote user moves outside the working range of the robotic arm, the reconstructed 3D model can continuously provide the user with a view of the current angle.

On the local-user side, the avatar and visual cues displayed by augmented reality can also effectively improve the interaction experience of local users. The avatar agents of remote users on the local end can make local users feel that they are no longer interacting with remote users that they cannot meet but are closer to natural face-to-face communication, and visual cues such as rays can also more clearly communicate the intentions of the remote users, thus improving the efficiency of interaction.

We can add richer animations to the avatar, e.g., by using speech-driven avatar mouth patterns [61] or by adding captured facial expressions [62,63,64]. In terms of nonverbal cues, we can consider using spatial mapping and tracking features in local space [3] or adding collision boxes to the reconstructed 3D model to make the virtual rays point more accurately at selected objects or surfaces.

### 6.4. Limitations

Our work has some limitations. Regarding view-sharing technology, due to the limitations of the equipment and network transmission conditions, we abandoned the solution of using a robotic arm to carry a 360 camera to move the view, as well as the solution of using 360 video as a view enhancement; regarding view enhancement, due to factors such as the field of view and distortion of the 3D camera, there is a certain degree of mismatch between the 3D video and the 3D model, which affects the viewing experience of remote users; regarding the evaluation of the system, due to some instability on the local-user side, we lacked objective metrics for local users and only used user studies with subjective interviews.

## 7. Conclusions

In this paper, we propose a robotic-arm-based movable telepresence system that allows remote users to actively control the view of the local space by moving their heads to observe the local environment more naturally. We then propose a 3D reconstruction method combined with stereoscopic video for a field-of-view enhancement that guides remote users to move within the range of movement of the robotic arm and provides them with a larger range of local environment perception. We also conducted a user study comparing our controllable 3D view with traditional 360 video and first-person view-sharing schemes in room-scale remote collaboration; the results showed that our system has significant advantages in efficiency, usability, coexistence, and perceptual information understanding over the other two views, and it was preferred by most participants. Additionally, we evaluated the entire system prototype through a user study. The study provided new design recommendations and directions for the use of movable 3D perspectives in mixed-reality telecollaboration and demonstrated the advantages of the system in a number of scenarios. In future work, we aim to improve the system’s design by integrating multiple-view-sharing solutions that provide users with the flexibility to switch between different views according to their individual needs. We also plan to conduct more extensive user studies to achieve additional enhancements. Specifically, we aim to fully leverage the capabilities of robotic arms, which enable remote users to impact the local environment, thereby improving collaboration efficiency and enhancing the interaction experience between remote and local users.

## Figures and Tables

**Table 1:** Results for the pretest and posttest in the SSQ under three conditions (*: *p* < 0.05, **: *p* < 0.01).

Techniques	Pre-SSQ-T		Pre-SSQ-T			
	Mean	SD	Mean	SD	Z	*p*
First-person view	6.39	11.16	15.58	22.73	74	0.006 **
360 video	5.61	14.59	12.16	28.06	92	0.012 *
Controllable 3D view	4.21	7.09	11.53	15.75	86	0.005 **

**Table 2:** Results of SSQ.

Techniques	N		O		D		T	
	Mean	SD	Mean	SD	Mean	SD	Mean	SD
First-person view	4.77	9.33	7.89	14.22	12.76	26.26	9.19	12.85
360 video	5.96	12.83	4.74	13.93	6.96	15.89	3.43	7.95
Controllable 3D view	5.17	11.16	4.74	10.44	11.02	21.32	7.32	13.16

**Table 3:** Advantages and disadvantages of the three view-sharing technologies in our system.

	Advantages	Disadvantages
First-person view	More intuitive view; no need to change orientation; smoother issuance of commands.	Changing perspective requires communication with the partner; not easy to search and observe the whole environment; unable to see the partner.
360 video	Widest viewing angle, least vertigo, and smoothest movement when turning the viewing angle.	High latency; insufficient screen resolution; inability to pan the view to observe an obscured area.
Controllable 3D view	Ability to actively move the viewpoint; best sense of immersion; allows for multiangle viewing of the workspace.	If the user moves too quickly, the screen will not be able to keep up, causing dizziness.

## Data Availability

Not applicable.

## Supplementary Materials

The following supporting information can be downloaded at: https://www.mdpi.com/article/10.3390/s23084113/s1, Video S1: Viewpoint controllable Telepresence: A robotic arm-based mixed-reality telecollaboration system.
